# Digital literacy and creative teaching behavior among nursing educators: the chain mediating roles of self-efficacy and digital stress

**DOI:** 10.3389/fpsyg.2026.1849999

**Published:** 2026-06-25

**Authors:** Wu Chong-Wen, Song Jin, Shasha Li, Zou Xiaoyue, Qu Baifeng, Wang Rui

**Affiliations:** 1 Department of Medical, HuZhou University, Huzhou, Zhejiang, China; 2 Intensive Care Unit, The First People's Hospital of Huzhou, Huzhou, Zhejiang, China; 3 Quality Control Office, Heilongjiang Provincial Hospital, Harbin, Heilongjiang, China; 4 Department of Nursing, Huzhou College, Huzhou, Zhejiang, China

**Keywords:** chain mediation, creative teaching behavior, digital literacy, digital stress, nursing educators, self-efficacy

## Abstract

**Background:**

The rapid digital transformation of nursing education has placed increasing demands on nurse educators, yet the psychological mechanisms linking digital literacy to creative teaching behavior remain poorly understood.

**Objectives:**

This study aimed to examine the chain mediating roles of self-efficacy and digital stress in the relationship between digital literacy and creative teaching behavior among nursing educators.

**Methods:**

A cross-sectional survey design was employed. Data were collected from 456 nursing educators recruited from six nursing colleges and universities in China. Participants completed validated self-report measures of digital literacy, self-efficacy, digital stress, and creative teaching behavior. Descriptive statistics, Pearson correlation analysis, and chain mediation analysis using the PROCESS macro (Model 6) with bootstrapping were conducted *via* SPSS 26.0.

**Results:**

Digital literacy was positively correlated with self-efficacy and creative teaching behavior, and negatively correlated with digital stress (all *p* < 0.001). Chain mediation analysis indicated that digital literacy influenced creative teaching behavior both directly (β = 0.231, *p* < 0.001) and indirectly *via* three significant pathways: through self-efficacy alone, through digital stress alone, and through the chain of self-efficacy → digital stress (all 95% CIs excluded zero).

**Conclusions:**

Self-efficacy and digital stress sequentially mediate the relationship between digital literacy and creative teaching behavior among nursing educators. Interventions targeting these psychological mechanisms may help promote creative teaching in the digital era.

## 1 Introduction

The global digital transformation of healthcare and higher education has reshaped the professional landscape for nursing educators (Jobst et al., 2022; Tischendorf et al., 2024a). As frontline practitioners of nursing pedagogy, nurse educators are expected not only to master digital tools for instruction but also to leverage them creatively to foster

clinical reasoning, simulation-based learning, and student engagement (Ghasemi et al., 2020; Pérez-Perdomo and Zabalegui, 2023). Creative teaching behavior, defined as the capacity of teachers to generate and implement instructional strategies that are novel, adaptive, and responsive to context, has emerged as a critical competency in contemporary nursing education, directly influencing students' learning outcomes and professional development (Chan, 2013; El-Sayed et al., 2025; Liu et al., 2020).

Digital literacy, broadly defined as the awareness, attitudes, and abilities of individuals to appropriately use digital tools and facilities to identify, access, manage, integrate, evaluate, and synthesize digital information, has been recognized as a foundational prerequisite for effective teaching in educational environments enriched by technology (Li et al., 2025; Nes et al., 2021; Tischendorf et al., 2024b). The inaugural World Conference on Digital Education underscored the urgency of enhancing educators' digital literacy as a central strategic imperative. In the nursing education context specifically, digital literacy equips nurse educators to design simulation-based curricula, utilize electronic health record systems pedagogically, and engage students through immersive digital platforms (Amin et al., 2025; Jobst et al., 2022). Empirical evidence has consistently demonstrated a positive association between digital literacy and creative thinking and teaching innovation (Asal et al., 2025; El-Sayed et al., 2025).

However, the pathway through which digital literacy translates into creative teaching behavior is unlikely to be direct. Two psychological mechanisms warrant particular theoretical attention. First, self-efficacy, defined as an individual's belief in their capacity to execute the behaviors necessary to produce specific outcomes, is recognized as a critical mediating variable (Ibrahim and Aldawsari, 2023). Teachers with higher digital literacy tend to exhibit elevated self-efficacy beliefs regarding their technological capabilities, and self-efficacy in turn is a robust predictor of creative teaching performance (Xiong et al., 2019). High self-efficacy empowers nurse educators to attempt novel instructional approaches, persist through pedagogical challenges, and invest cognitive effort in creative lesson design (Alibakhshi et al., 2020).

The intensification of technology use in nursing education has introduced a paradoxical burden: digital stress (also termed technostress), defined as the psychological strain arising from an individual's inability to adapt to digital technologies in a healthy manner (Brod, 1984). Digital stress encompasses dimensions of techno-overload, techno-complexity, techno-insecurity, techno-invasion, and techno-uncertainty (Ragu-Nathan et al., 2008). Research has consistently demonstrated that higher self-efficacy serves as a protective factor against digital stress (Zivi et al., 2025), and that elevated digital stress in turn impairs creative and innovative teaching behaviors through cognitive resource depletion and increased resistance to technological innovation (Zhang, 2023).

Grounded in Bandura's Social Cognitive Theory (Bandura, 2001) and the Job Demands-Resources (JD-R) model (Bakker and Demerouti, 2017), we propose that digital literacy enhances nursing educators' self-efficacy, which in turn reduces their experience of digital stress, ultimately promoting creative teaching behavior. Self-efficacy occupies a pivotal position in this model because it carries a distinct meaning in each framework. In SCT, self-efficacy is the agentic mechanism through which competence is translated into motivated, innovative action; in the JD-R model, it functions as a personal resource that buffers job demands. Digital literacy can thus be understood as a mastery-based resource that both strengthens efficacy beliefs and, through them, reshapes how technological demands are appraised: educators with stronger efficacy beliefs are more likely to construe digital demands as manageable challenges rather than threats, and accordingly experience lower digital stress. The JD-R health-impairment pathway then specifies why this matters for creativity, as unmitigated digital stress depletes the cognitive resources required for novel pedagogical work. The hypothesized chain—digital literacy → self-efficacy → digital stress → creative teaching behavior—follows from the convergence of SCT's account of how efficacy beliefs govern appraisal and action with the JD-R account of how demands and resources shape work behavior. This chain mediation framework has not been empirically tested in the nursing educator population, representing a significant gap in the literature.

The specific aims of this study were to: (1) describe the levels of digital literacy, self-efficacy, digital stress, and creative teaching behavior among nursing educators; (2) examine the bivariate correlations among these four variables; and (3) test the chain mediation model in which self-efficacy and digital stress sequentially mediate the relationship between digital literacy and creative teaching behavior.

## 2 Methods

### 2.1 Study design and setting

A cross-sectional, correlational survey design was employed. Participants were recruited from six nursing colleges and universities located across four provinces in China. The study was conducted and reported in accordance with the STROBE guidelines for observational studies.

### 2.2 Participants

Nursing educators were eligible to participate if they: (1) held a formal teaching position at a nursing college or university; (2) were actively engaged in undergraduate or postgraduate nursing instruction; and (3) provided written informed consent. Educators on extended leave or who had not used any digital teaching tools in the preceding 3 months were excluded.

Participants were recruited through purposive sampling. Given the complexity of the chain mediation model involving two sequential mediators and multiple covariates, more conservative recommendations of at least 300–500 participants have been proposed for stable bootstrap estimation of indirect effects in multiple mediator models. Additionally, accounting for an anticipated invalid response rate of approximately 10%−15%, a minimum recruitment target of 353 participants was established prior to data collection. A total of 462 questionnaires were distributed, of which six were excluded due to non-differentiating responses, yielding a final valid sample of 456 participants (valid response rate = 98.7%).

### 2.3 Measures

#### 2.3.1 Digital literacy

Digital literacy was assessed using the College Teacher Digital Literacy Scale developed by Liu et al., comprising 32 items across five dimensions: digital awareness, digital technology knowledge and skills, digital application, digital social responsibility, and professional development (Liu Xinyi and Yuan, 2025). Items were rated on a 5-point Likert scale ranging from 1 (completely disagree) to 5 (completely agree), with higher scores indicating greater digital literacy. This scale has demonstrated good reliability and validity in prior studies, with Cronbach's α of 0.978. In the present sample, Cronbach's α was 0.913.

#### 2.3.2 Teacher self-efficacy

Teacher self-efficacy was measured using the short-form Teachers' Sense of Efficacy Scale (TSE) developed by Tschannen-Moran et al. (1998), as translated and adapted into Chinese by Wu Liang (2017). The scale comprises 12 items across two dimensions: efficacy for classroom management and efficacy for instructional strategies and student engagement. Items were rated on a 5-point Likert scale ranging from 1 (strongly disagree) to 5 (strongly agree), with higher scores reflecting a stronger sense of professional self-efficacy. A sample item reads: “I am able to stop disruptive behavior in the classroom.” In the present sample, Cronbach's α was 0.876.

#### 2.3.3 Digital stress

Digital stress was assessed using the Technostress Creators Scale originally developed by Tarafdar et al. (2007) and subsequently revised by Jiang to better reflect the characteristics of the teacher population (Prior et al., 2016). The scale comprises 23 items across five dimensions: techno-overload, techno-invasion, techno-complexity, techno-insecurity, and techno-uncertainty. Items were rated on a 5-point Likert scale ranging from 1 (completely disagree) to 5 (completely agree), with higher total scores indicating greater levels of perceived digital stress. In the present sample, Cronbach's α was 0.891.

#### 2.3.4 Creative teaching behavior

Creative teaching behavior was measured using the Creativity Fostering Teacher Index originally developed by Cropley (1997) and revised for the Chinese educational context by Zhang et al. (2008). The scale comprises 28 items across four dimensions: guidance of learning approaches, motivation stimulation, evaluation of perspectives, and encouragement of flexible thinking. Items were rated on a 5-point Likert scale ranging from 1 (completely disagree) to 5 (completely agree), with higher mean scores indicating a higher level of creative teaching behavior. In the present sample, Cronbach's α was 0.898.

### 2.4 Data collection

Data were collected between February and March 2026 using the Chinese online survey platform Wenjuanxing (www.wjx.cn), which is functionally analogous to Qualtrics and widely used in educational research in China. Invitations were distributed *via* WeChat groups, along with a brief description of the study's purpose and an assurance of voluntary participation and data confidentiality. To ensure response quality, attention-check items were embedded within the questionnaire, and submissions completed in an implausibly short time (i.e., less than 2 min) were excluded from the final dataset. After excluding incomplete responses and those failing the attention checks, 456 valid responses were retained for analysis, yielding a valid response rate of 98.7%.

### 2.5 Data analysis

All statistical analyses were performed using IBM SPSS Statistics 26.0 (IBM Corp., Armonk, NY, USA) and the PROCESS macro version 4.3. Descriptive statistics (means, standard deviations, and ranges) and Pearson correlation coefficients were computed for all study variables. The assumptions of normality and multicollinearity were verified prior to mediation analysis.

The chain mediation model was tested using PROCESS Model 6. Bootstrapping with 5,000 resamples and 95% bias-corrected confidence intervals (BCIs) was employed to estimate indirect effects (Prior et al., 2016). An indirect effect was considered statistically significant when the 95% BCI did not include zero. Age, sex, educational level, years of teaching experience, and professional title were entered as covariates in all models. Statistical significance was set at *p* < 0.05 (two-tailed).

### 2.6 Ethical considerations

This study was approved by the Ethics Committee of Huzhou University. All participants provided written informed consent prior to participation. Data were anonymized and stored securely in compliance with institutional data governance policies. Participation was entirely voluntary, and participants were informed of their right to withdraw at any time without consequence.

## 3 Result

### 3.1 Common method bias

Given that all measures were self-reported and collected simultaneously, two complementary diagnostic procedures were used to assess common method bias. First, Harman's single-factor test was conducted. The unrotated principal component analysis revealed that the first factor accounted for 17.82% of the total variance, well below the recommended threshold of 50%, indicating that common method bias was unlikely to constitute a serious threat. Second, following Kock (2015), a full collinearity test was conducted by regressing all study variables on a common method factor; all variance inflation factors (VIFs) ranged from 1.30 to 1.55, well below the conservative threshold of 3.3, providing further evidence that common method bias was unlikely to substantially distort the findings.

### 3.2 Measurement model and discriminant validity

Confirmatory factor analysis (CFA) was conducted to examine the discriminant validity of the four constructs. The hypothesized four-factor model demonstrated good fit to the data, χ^2^/df = 1.08, CFI = 0.967, TLI = 0.966, RMSEA = 0.014, SRMR = 0.040. This model fit substantially better than a series of constrained alternative models, including a three-factor model combining the two mediators (CFI = 0.859), a two-factor model (CFI = 0.747), and a one-factor model (CFI = 0.626), supporting the distinctiveness of the four constructs. Composite reliability was acceptable for all constructs (CR = 0.877–0.913), and all heterotrait–monotrait (HTMT) ratios fell well below the 0.85 criterion (range 0.39–0.55), providing further support for discriminant validity among digital literacy, self-efficacy, digital stress, and creative teaching behavior.

### 3.3 Participant characteristics

A total of 456 nursing educators participated in this study. The majority were female (*n* = 380, 83.3%), with male educators comprising 16.7% (*n* = 76). In terms of age, 30.5% (*n* = 139) were aged ≤ 35 years, 37.3% (*n* = 170) were aged 36–49 years, and 32.2% (*n* = 147) were aged ≥50 years. Regarding teaching experience, participants were relatively evenly distributed across four groups: ≤ 5 years (24.1%, *n* = 110), 6–10 years (24.1%, *n* = 110), 11–20 years (24.8%, *n* = 113), and >20 years (27.0%, *n* = 123). With respect to professional title, 24.8% held junior (*n* = 113), 48.9% intermediate (*n* = 223), and 26.3% senior (*n* = 120) titles. Most participants had no administrative responsibilities (88.2%, *n* = 402). In terms of educational attainment, 30.9% held a bachelor's degree (*n* = 141), 43.2% a master's degree (*n* = 197), and 25.9% a doctoral degree (*n* = 118).

One-way ANOVA revealed that professional title (*F* = 14.028, *p* < 0.001) and educational level (*F* = 18.239, *p* < 0.001) significantly differentiated digital literacy scores, with intermediate-titled educators (*M* = 3.135) and master's degree holders (*M* = 3.172) reporting the highest levels. Administrative role (*F* = 3.866, *p* = 0.050) and age group (*F* = 3.016, *p* = 0.050) also showed marginal effects. Gender (*F* = 0.057, *p* = 0.812) and teaching experience (*F* = 0.533, *p* = 0.660) did not significantly differentiate digital literacy. For creative teaching behavior, professional title (*F* = 12.126, *p* < 0.001) and educational level (*F* = 5.670, *p* = 0.004) were significant differentiating factors; the remaining demographic variables did not yield significant group differences (all *p* > 0.05).

### 3.4 Descriptive statistics and correlations

Prior to the main analyses, the distributional properties of the study variables were examined. Skewness values ranged from −0.14 to 0.09 and kurtosis values from −0.46 to −0.26, all within the ±1 criterion, supporting the assumption of univariate normality.

Descriptive statistics and Pearson correlations among the study variables are presented in Table 1. Digital literacy (*M* = 96.07, SD = 17.47, α = 0.913), self-efficacy (*M* = 36.04, SD = 8.24, α = 0.877), digital stress (*M* = 68.93, SD = 13.10, α = 0.892), and creative teaching behavior (*M* = 69.04, SD = 13.34, α = 0.898) all demonstrated acceptable to good internal consistency.

**Table 1:** Descriptive statistics and Pearson correlations among study variables (*N* = 456).

Variable	M ±SD	Digital literacy	Self-efficacy	Digital stress
Digital literacy	96.07 ± 17.47	1		
Self-efficacy	36.04 ± 8.24	0.484^***^	1	
Digital stress	68.93 ± 13.10	−0.410^***^	−0.389^***^	1
Creative teaching behavior	69.04 ± 13.34	0.441^***^	0.492^***^	−0.343^***^

^***^p < 0.001.

Bivariate correlations indicated that digital literacy was positively associated with self-efficacy (*r* = 0.484, *p* < 0.001) and creative teaching behavior (*r* = 0.441, *p* < 0.001), and negatively associated with digital stress (*r* = −0.410, *p* < 0.001). Self-efficacy was positively correlated with creative teaching behavior (*r* = 0.492, *p* < 0.001) and negatively correlated with digital stress (*r* = −0.389, *p* < 0.001). Digital stress was negatively correlated with creative teaching behavior (*r* = −0.343, *p* < 0.001). These patterns are consistent with the theoretical framework underlying the hypothesized chain mediation model.

### 3.5 Chain mediation analysis

**Figure 1:**
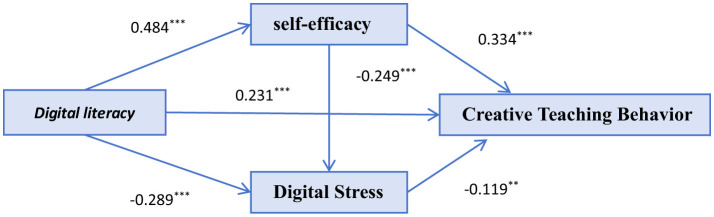
The chain mediation model with standardized path coefficients. *Note:* **p < 0.01, ***p < 0.001.

To examine whether self-efficacy and digital stress sequentially mediated the relationship between digital literacy and creative teaching behavior, a chain mediation model (PROCESS Macro, Model 6; Hayes, 2022) was estimated with 5,000 bootstrap resamples and 95% bias-corrected confidence intervals. All variables were standardized prior to analysis. The standardized path coefficients are presented in Table 2, the decomposition of total, direct, and indirect effects is reported in Table 3, and the model is illustrated in Figure 1.

**Table 2:** Standardized path coefficients and decomposition of effects in the chain mediation model.

Effect pathway	β	SE	*t*	*P*
Digital literacy → self-efficacy	0.484	0.041	11.79	< 0.001
Digital literacy → digital stress	−0.289	0.048	−6.08	< 0.001
Self-efficacy → digital stress	−0.249	0.048	−5.24	< 0.001
Self-efficacy → creative teaching behavior	0.334	0.046	7.24	< 0.001
Digital stress → creative teaching behavior	−0.119	0.044	−2.68	0.008
Digital literacy → creative teaching behavior	0.231	0.047	4.95	< 0.001

**Table 3:** Decomposition of total, direct, and indirect effects.

Effect pathway	β	SE	95% CI LL	95% CI UL
Total effect (X → Y)	0.441	0.042	0.380	0.557
Direct effect (X → Y)	0.231	0.044	0.143	0.315
Total indirect effect	0.210	0.028	0.158	0.269
Indirect path 1: X → M1 → Y	0.162	0.024	0.119	0.212
Indirect path 2: X → M2 → Y	0.034	0.014	0.010	0.064
Indirect path 3: X → M1 → M2 → Y (chain)	0.014	0.007	0.004	0.030

X = Digital Literacy; M1 = Self-Efficacy; M2 = Digital Stress; Y = Creative Teaching Behavior. Bootstrap n = 5,000. CI = confidence interval; LL = lower limit; UL = upper limit. Confidence intervals not containing zero indicate significant indirect effects.

As shown in Table 1, digital literacy was significantly associated with self-efficacy (path a1: β = 0.484, SE = 0.041, *t* = 11.79, *p* < 0.001) and negatively associated with digital stress (path a2: β = −0.289, SE = 0.048, *t* = −6.08, *p* < 0.001). Controlling for digital literacy, self-efficacy was negatively associated with digital stress (path d21: β = −0.249, SE = 0.048, *t* = −5.24, *p* < 0.001). In the outcome equation, self-efficacy was significantly and positively associated with creative teaching behavior (path b1: β = 0.334, SE = 0.046, *t* = 7.24, *p* < 0.001), and digital stress was significantly and negatively associated with creative teaching behavior (path b2: β = −0.119, SE = 0.044, *t* = −2.68, *p* = 0.008). The model explained 23.4% of the variance in self-efficacy, 21.6% in digital stress, and 30.6% in creative teaching behavior.

Table 2 summarizes the decomposition of effects on creative teaching behavior. The total effect of digital literacy on creative teaching behavior was significant (β = 0.441, SE = 0.042, *t* = 10.46, *p* < 0.001). After entering self-efficacy and digital stress simultaneously, the direct effect of digital literacy on creative teaching behavior remained significant [β = 0.231, SE = 0.044, 95% CI (0.143, 0.315)], indicating partial mediation. The total indirect effect was also significant [Effect = 0.210, SE = 0.028, 95% CI (0.158, 0.269)].

Three specific indirect pathways were examined. Indirect Path 1 (digital literacy → self-efficacy → creative teaching behavior) was significant [Effect = 0.162, SE = 0.024, 95% CI (0.119, 0.212)], supporting mediation through self-efficacy. Indirect Path 2 (digital literacy → digital stress → creative teaching behavior) was also significant [Effect = 0.034, SE = 0.014, 95% CI (0.010, 0.064)]) supporting mediation through digital stress. Indirect Path 3 (digital literacy → self-efficacy → digital stress → creative teaching behavior), the hypothesized chain mediation pathway, was significant [Effect = 0.014, SE = 0.007, 95% CI (0.004, 0.030)].

## 4 Discussion

This study examined the chain mediating roles of self-efficacy and digital stress in the relationship between digital literacy and creative teaching behavior among nursing educators. The results supported all hypothesized pathways. Digital literacy was positively associated with creative teaching behavior, and this relationship was partially mediated through three significant indirect pathways: *via* self-efficacy alone, *via* digital stress alone, and *via* the sequential chain of self-efficacy followed by digital stress. The direct effect of digital literacy on creative teaching behavior remained significant after the mediators were entered, indicating partial rather than full mediation. Taken together, these findings advance our understanding of the psychological mechanisms potentially linking digital competence with pedagogical creativity in the nursing education context.

### 4.1 Digital literacy and creative teaching behavior

The significant positive total effect of digital literacy on creative teaching behavior is consistent with and extends prior literature demonstrating that digital competence is positively associated with creative thinking and innovative instructional practices (Voogt, 2012). Nursing educators with higher digital literacy possess broader repertoires of technological tools and pedagogical strategies, affording them the flexibility and cognitive scaffolding necessary for creative instructional design (Cayirdag, 1959). This finding aligns with the Digital Competence Framework for Educators, which explicitly situates creative problem-solving and pedagogical innovation as advanced-level digital competency outcomes (Li et al., 2025). Importantly, the persistence of a significant direct effect after controlling for both mediators suggests that digital literacy influences creative teaching behavior both through psychological pathways and through additional mechanisms not captured in the present model, such as direct access to a broader range of digital teaching tools and resources that enable creative instructional design independently of psychological states.

### 4.2 The mediating role of self-efficacy

Self-efficacy was the strongest single mediator in the model, accounting for an indirect effect of 0.162, representing approximately 77% of the total indirect effect. This finding is theoretically grounded in Bandura's Social Cognitive Theory (Bandura, 2001), which posits that actual competence must be accompanied by efficacy beliefs to be translated into motivated, innovative behavior. The present results confirm that digital literacy significantly predicted self-efficacy, and that self-efficacy in turn significantly predicted creative teaching behavior. These findings corroborate prior research demonstrating that digital literacy is a significant positive predictor of technology-related self-efficacy (Prior et al., 2016; Tabaru Örnek, 2026) and that self-efficacy is positively associated with creative teaching (Cayirdag, 1959; Xiong et al., 2019).

The particularly strong a1 path coefficient is noteworthy and suggests that among nursing educators, the experience of digital competence is a potent source of efficacy information-consistent with Bandura's proposition that mastery experience constitutes the most influential source of self-efficacy beliefs (Xiong et al., 2019). Nursing educators who have developed proficiency in navigating digital tools, designing online learning environments, and managing digital health information systems may derive from these experiences a generalized confidence that empowers them to attempt novel and creative pedagogical strategies. In this sense, digital literacy may function not merely as a technical resource but as a psychological correlate of instructional innovation.

### 4.3 The mediating role of digital stress

Digital stress emerged as a significant, albeit weaker, independent mediator. Digital literacy negatively predicted digital stress, and digital stress in turn negatively predicted creative teaching behavior. These findings are consistent with the Job Demands-Resources (JD-R) model (Bakker and Demerouti, 2017), which conceptualizes digital stress as a job demand that depletes the psychological and cognitive resources necessary for proactive and creative work behavior. Nursing educators with higher digital literacy may be better equipped to navigate the complexity and uncertainty of digitalized teaching environments, including the demands of working across professional and disciplinary boundaries, and accordingly report lower levels of digital stress and greater cognitive resources for creative instructional engagement (Califf and Brooks, 2020; Li and Wang, 2021).

The comparatively modest effect size of the digital stress indirect pathway relative to the self-efficacy pathway warrants consideration. One possible interpretation is that digital stress operates primarily as a suppressor of creative behavior rather than as an independent driver of creativity; that is, its influence is most pronounced when other psychological resources, particularly self-efficacy, are depleted. This interpretation is supported by the chain mediation findings discussed below.

### 4.4 The chain mediation

The core theoretical contribution of this study lies in the confirmation of the sequential chain mediation pathway. Although the absolute effect size of this chain pathway was modest, its confidence interval excluded zero, confirming statistical significance, and its theoretical significance is substantial. The chain pathway articulates a coherent and previously untested psychological mechanism: higher digital literacy is associated with nurse educators' stronger sense of competence and mastery, which in turn is linked to a lower appraisal of digital demands as threatening and cognitively overwhelming, and consequently with greater availability of psychological resources for creative pedagogical engagement.

This sequential mechanism integrates Social Cognitive Theory's emphasis on efficacy beliefs as translators of competence into behavior with the JD-R model's conceptualization of psychological resources as buffers against occupational demands. The two theories contribute distinct but complementary levels of explanation. Social Cognitive Theory explains why efficacy beliefs reduce strain at the level of individual appraisal: educators high in self-efficacy construe technological demands as manageable challenges rather than threats and sustain coping effort under difficulty. The JD-R model, in turn, specifies the functional role of that same relationship within a system of work design, where self-efficacy operates as a personal resource that dampens a job demand. SCT supplies the psychological micro-mechanism for a relationship that JD-R describes structurally, and JD-R locates that SCT prediction within an occupational demand–resource framework. The chain pathway observed here is thus consistent with both accounts operating on the same set of constructs. In concrete terms, the chain suggests that digital literacy training alone may be insufficient to support creative teaching behavior if it does not concurrently build educators' efficacy beliefs; and that efficacy beliefs may matter not only directly for creative behavior but also indirectly by being associated with lower digital stress. This nuanced, sequential account offers a more mechanistic explanation of the digital literacy–creative teaching behavior relationship than any single-mediator model could provide, and represents a novel empirical contribution to both nursing education and educational psychology literature.

The finding also has important implications for understanding why some digitally literate educators nevertheless fail to exhibit creative teaching behavior. The chain model suggests that such educators may possess technical skills while still experiencing high digital stress, perhaps because their efficacy beliefs have not been adequately consolidated through supported mastery experiences, and that this stress burden may be associated with reduced creative pedagogical output.

### 4.5 Practical implications

The findings, while correlational, suggest several directions that may inform future intervention research. Because the proposed mechanisms have not yet been causally established, the following implications should be regarded as hypotheses to be tested rather than as prescriptions derived directly from the present data.

Given the prominent mediating role observed for self-efficacy, one promising direction is to examine whether digital literacy training that incorporates efficacy-building components, such as supervised low-stakes practice, peer observation, and mentoring schemes that pair senior with junior educators, translates digital competence into creative teaching behavior more effectively than technical upskilling alone. Such possibilities would need to be tested experimentally before firm recommendations could be made.

Similarly, the association between digital stress and creative teaching behavior raises the question of whether organizational measures to mitigate digital stress, such as dedicated technical support, workload boundaries around digital availability, and peer support networks, might help preserve educators' capacity for creative teaching. Whether such measures would yield the hypothesized benefits remains an empirical question for future longitudinal or intervention studies.

Finally, the demographic patterns observed here tentatively suggest that junior-titled educators may be a relevant group for targeted support, given their potentially greater vulnerability to the combined burden of lower digital literacy, attenuated self-efficacy, and elevated digital stress. This proposition, too, would require prospective testing before informing the design of stratified faculty development programs.

### 4.6 Limitations

Several limitations should be acknowledged. The cross-sectional design precludes causal inference, and longitudinal or experimental research is needed to confirm the temporal ordering of the proposed chain. Reliance on self-report measures introduces potential social desirability bias. Although both Harman's single-factor test and the full collinearity VIF analysis suggested that common method bias was unlikely to be a serious confound, these *post-hoc* statistical checks cannot fully rule out method-related variance. Future studies should adopt procedural remedies, such as collecting data from multiple sources (e.g., peer or supervisor ratings of creative teaching behavior), separating measurement of predictors and outcomes in time, or employing experimental designs to more rigorously address this issue. The sampling frame was restricted to nursing educators in China, limiting generalizability to other national contexts. In addition, self-efficacy was assessed with a general teacher self-efficacy scale rather than a technology-specific measure, creating a gap between our domain-relevant theoretical argument and its operationalization. Because efficacy beliefs are most predictive when matched to the target domain, this likely attenuated the observed associations, rendering our tests conservative. Future studies should adopt a digital teaching self-efficacy measure. Finally, the modest effect size of the chain indirect pathway suggests that additional variables, such as organizational support, intrinsic motivation, or emotional regulation -may account for further variance and warrant inclusion in future models.

## 5 Conclusions

This study identified a significant chain mediation model in which self-efficacy and digital stress sequentially mediated the relationship between digital literacy and creative teaching behavior among 456 nursing educators. Beyond a significant direct effect, digital literacy was associated with creative teaching indirectly through stronger self-efficacy, lower digital stress, and through the sequential pathway of both mediators. These findings, integrating Social Cognitive Theory with the Job Demands-Resources model, suggest that institutional efforts to foster creative nursing pedagogy should extend beyond digital skills training to simultaneously support educators' efficacy beliefs and address technology-induced occupational strain.

## Data Availability

The datasets presented in this article are not readily available because “The data that support the findings of this study are available on request from the corresponding author”. Requests to access the datasets should be directed to wcw202506@163.com.
